# How Food Marketing on Instagram Shapes Adolescents’ Food Preferences: Online Randomized Trial

**DOI:** 10.2196/28689

**Published:** 2021-10-22

**Authors:** Marie Bragg, Samina Lutfeali, Tenay Greene, Jessica Osterman, Madeline Dalton

**Affiliations:** 1 Department of Population Health New York University Grossman School of Medicine New York City, NY United States; 2 Stanford Graduate School of Business Palo Alto, CA United States; 3 Department of Pediatrics Dartmouth Geisel School of Medicine Hanover, NH United States

**Keywords:** food marketing, traditional media, social media, adolescents, Instagram

## Abstract

**Background:**

Worldwide obesity rates have prompted 16 countries to enact policies to reduce children’s exposure to unhealthy food marketing, but few policies address online advertising practices or protect adolescents from being targeted. Given adolescents spend so much time online, it is critical to understand how persuasive Instagram food advertisements (ads) are compared with traditional food ads. To strengthen online food marketing policies, more evidence is needed on whether social media ads are more persuasive than other types of ads in shaping adolescents’ preferences.

**Objective:**

This study examined whether adolescents could identify food companies’ Instagram posts as ads, and the extent to which Instagram versus traditional food ads shape adolescents’ preferences.

**Methods:**

In Part 1, participants aged 13-17 years (N=832) viewed 8 pairs of ads and were asked to identify which ads originated from Instagram. One ad in each pair was selected from traditional sources (eg, print; online banner ad), and the other ad was selected from Instagram, but we removed the Instagram frame—which includes the logo, comments, and “likes.” In Part 2, participants were randomized to rate food ads that ostensibly originated from (1) Instagram (ie, we photoshopped the Instagram frame onto ads); or (2) traditional sources. Unbeknownst to participants, half of the ads in their condition originated from Instagram and half originated from traditional sources.

**Results:**

In Part 1, adolescents performed worse than chance when asked to identify Instagram ads (*P*<.001). In Part 2, there were no differences on 4 of 5 outcomes in the “labeled ad condition.” In the “unlabeled ad condition,” however, they preferred Instagram ads to traditional ads on 3 of 5 outcomes (ie, trendiness, *P*=.001; artistic appeal, *P*=.001; likeability, *P*=.001).

**Conclusions:**

Adolescents incorrectly identified traditional ads as Instagram posts, suggesting the artistic appearance of social media ads may not be perceived as marketing. Further, the mere presence of Instagram features caused adolescents to rate food ads more positively than ads without Instagram features.

## Introduction

The high prevalence of childhood obesity persists in the United States, and the National Academy of Medicine identifies food marketing as a major contributing factor [1]. Despite both national and international efforts to restrict child-targeted food marketing, most policies do not protect adolescents aged 13-17 years, nor do they address social media advertising [2]. Yet adolescents spend an increasingly high number of hours online each day.

Recognizing social media’s growing popularity, food companies have dramatically increased their advertising presence on social media platforms in ways that appeal to young audiences. Coca-Cola, for example, dedicates 20% of their US $4 billion annual advertising budget to social media [3]. One descriptive study reported that 6.2 million adolescents followed a sample of 27 food and beverage brands on Instagram and Twitter [4]. This shift in marketing and exposure concerns public health experts [5] because studies have shown that food companies’ official social media accounts promote mostly unhealthy products [6-8], and increase brand recognition among youth [9,10].

Adolescents’ unique developmental stage may compound their vulnerability to social media food advertisements (ads). Social Norms Theory [11-13] suggests social media may capitalize on adolescents’ exquisite sensitivity to peer behavior—“likes,” for example, represent social norms that may signal to adolescents which social media accounts they should follow [14-16]. Experts have expressed concern that the ability to “like” posts also makes social media advertising uniquely interactive and may lead adolescents to perceive brands as friends more than companies [10]. Ads posted by companies may look similar to posts by friends because of context (ie, they appear interspersed with posts shared by friends) and content (ie, companies’ posts mimic the aesthetic of everyday consumers’ posts), and it is unclear whether these marketing strategies cloud adolescents’ ability to identify company posts as advertising. A neuroimaging study showed heightened activity in the nucleus accumbens—a reward hub of the brain—among adolescents who viewed their own posts with high numbers of “likes” versus few “likes” [17]. Given the nucleus accumbens is more sensitive to reward among adolescents compared with adults and children [18], those findings reinforce adolescents’ unique susceptibility to social media ads.

Despite the emerging research on adolescents’ engagement with food marketing on social media, no studies have compared the power of unhealthy food ads on social media versus more traditional ads (eg, print ads; noninteractive website banner ads). It is not known whether adolescents are skilled at identifying social media ads. Besides, no studies have examined whether social media advertising is more powerful than traditional ads in its ability to affect adolescents’ preferences. Finally, the combined effects of showing adolescents food ads and Instagram frames that include the Instagram logo, “likes,” and comments have not been explored. Instagram frames that include the Instagram logo, “likes,” and comments may have their own brand power and make ads highly appealing to adolescents.

The objective of this study is to address those gaps by examining whether adolescents could distinguish between food companies’ Instagram ads and their more traditional ads, and the extent to which Instagram food ads generate more appeal compared with traditional ads. Given adolescents’ frequent social media use [19], we hypothesized that participants would correctly identify Instagram ads, and that they would prefer Instagram ads more than traditional ads regardless of whether ads were photoshopped to appear as though they had originated from Instagram.

## Methods

### Study Population

We recruited 1044 adolescents aged 13-17 years who identified as either Black/African American or non-Latino White through Dynata, a firm that recruits research participants using online panels, digital networks, websites, SMS text messaging, and telephone alerts. The Institutional Review Board at New York University School of Medicine approved our study.

Of the 1044 adolescents who started the survey, 976 completed it, and 884 correctly answered our data integrity question (ie, “Type ‘Facebook’ in the box below.”). A total of 52 adolescents identified as a race/ethnicity other than Black/African American or non-Latino White and were excluded from the analyses. We included only Black and non-Latino White adolescents in our sample because companies disproportionately target Black adolescents with their least healthy products [20,21], and White adolescents are featured in the majority of food ads [22]. Because of these differences in targeted marketing, we designed stimuli that featured either Black or non-Latino White individuals. Secondary analyses that examine a subset of racially targeted ads are under review elsewhere. Among the final sample (N=832), 387 (46.5%) adolescents identified as Black or African American and 445 (53.5%) adolescents identified as non-Latino White. Table 1 presents adolescents’ self-reported demographic characteristics and social media usage.

**Table 1:** Demographic characteristics and social media usage of sample: January–June 2018.

Demographic characteristics		Total sample (N=832)	Part 2: Labeled advertisement condition (n=381)	Part 2: Unlabeled advertisement condition (n=451)
Age, years, mean, (SD)		14.73 (1.67)	14.75 (1.64)	14.71 (1.69)
**Gender, n (%)**				
	Male	426 (51.2)	197 (51.7)	229 (50.8)
	Female	406 (48.8)	184 (48.3)	222 (49.2)
**Race, n (%)**				
	Non-Latino White	445 (53.5)	211 (55.4)	234 (51.9)
	Black/African American	387 (46.5)	170 (44.6)	217 (48.1)
**When do you use social media?, n (%)**				
	Right when you wake up	305 (36.7)	131 (34.4)	174 (38.6)
	Before school	407 (48.9)	173 (45.4)	234 (51.9)
	On the way to school	309 (37.1)	143 (37.5)	166 (36.8)
	At school	258 (31.0)	115 (30.2)	143 (31.7)
	During lunch	381 (45.8)	173 (45.4)	208 (46.1)
	On the way home from school	323 (38.8)	151 (39.6)	172 (38.1)
	After school	381 (45.8)	257 (67.5)	307 (68.1)
	While doing homework	264 (31.7)	119 (31.2)	145 (32.2)
	After doing homework	390 (46.9)	180 (47.2)	210 (46.6)
	During dinner	158 (19.0)	74 (19.4)	84 (18.6)
	Before bed	497 (59.7)	232 (60.9)	265 (58.8)
	Right before going to sleep	234 (28.1)	102 (26.8)	132 (29.3)
**Social media account history, n (%)**				
	Do you have...[check all that apply]			
	Instagram?	582 (70.0)	279 (73.2)	303 (67.2)
	Facebook?	710 (85.3)	321 (84.3)	389 (86.3)
	Snapchat?	410 (49.3)	189 (49.6)	221 (49.0)
	Tumblr?	71 (8.5)	40 (10.5)	31 (6.9)
	Twitter?	394 (47.4)	194 (50.9)	200 (44.3)
Average number of social accounts per participant based on responses to question above, mean (SD)		2.60 (1.20)	2.69 (1.15)	2.54 (1.20)
Have you made a purchase through social media before?, n (%)		167 (20.1)	83 (21.8)	84 (18.6)

### Advertisement Development

We identified the 10 most advertised food and beverage brands in the United States [23] and then identified 10 analogous brands originating outside of the United States (eg, Lay’s Potato Chips from the United States vs. Walker’s Crisps from the UK). We identified those analogous brands because consumers’ familiarity with brands could potentially affect their survey responses [24]. We then aimed to select from those brands (1) 200 unhealthy food and beverage ads on Instagram; and (2) 200 traditional ads to serve as potential stimuli. To select the 200 Instagram ads for unhealthy food and beverages, we asked 10 research assistants to screen capture 10 Instagram ads from the official accounts of the most advertised brands and their analogous brands. Research assistants screen captured a random sample of 10 ads for unhealthy products from each brand (ie, 10 ads from 10 US brands + 10 ads from 10 analogous brands = 200 Instagram ads as potential stimuli). To select a random sample of 10 ads from each brand’s Instagram account, research assistants were instructed to use a random number generator and then select the ad corresponding to the generated number by counting from the most recently posted ad. For example, if the number “10” was randomly generated, the research assistant would select the tenth most recent ad posted to the brand’s Instagram feed. To identify traditional ads, we asked those same research assistants to capture 10 traditional ads for the same 10 most advertised and 10 analogous brands by searching on Google and AdScope, a repository of all ads from television, print, radio, and the internet [25].

To assess the pairings of traditional and Instagram ads, we generated a codebook of ad themes using an iterative process. Researchers identified ad themes by assessing the ad image and text (eg, snowflakes or references to the cold for a “winter” theme). They then searched for traditional and Instagram ads with matching themes. In our first review of the matched ad pairs, we met 90% agreement. Discrepancies were resolved by consensus. The final ad themes included the following: features a human actor, features a nonhuman character, race of actor product visibility, product handling (eg, whether a person or character in the image holds the promoted product), seasonal (eg, fall, winter, spring, and summer themes), and number of actors or characters.

**Figure 1:**
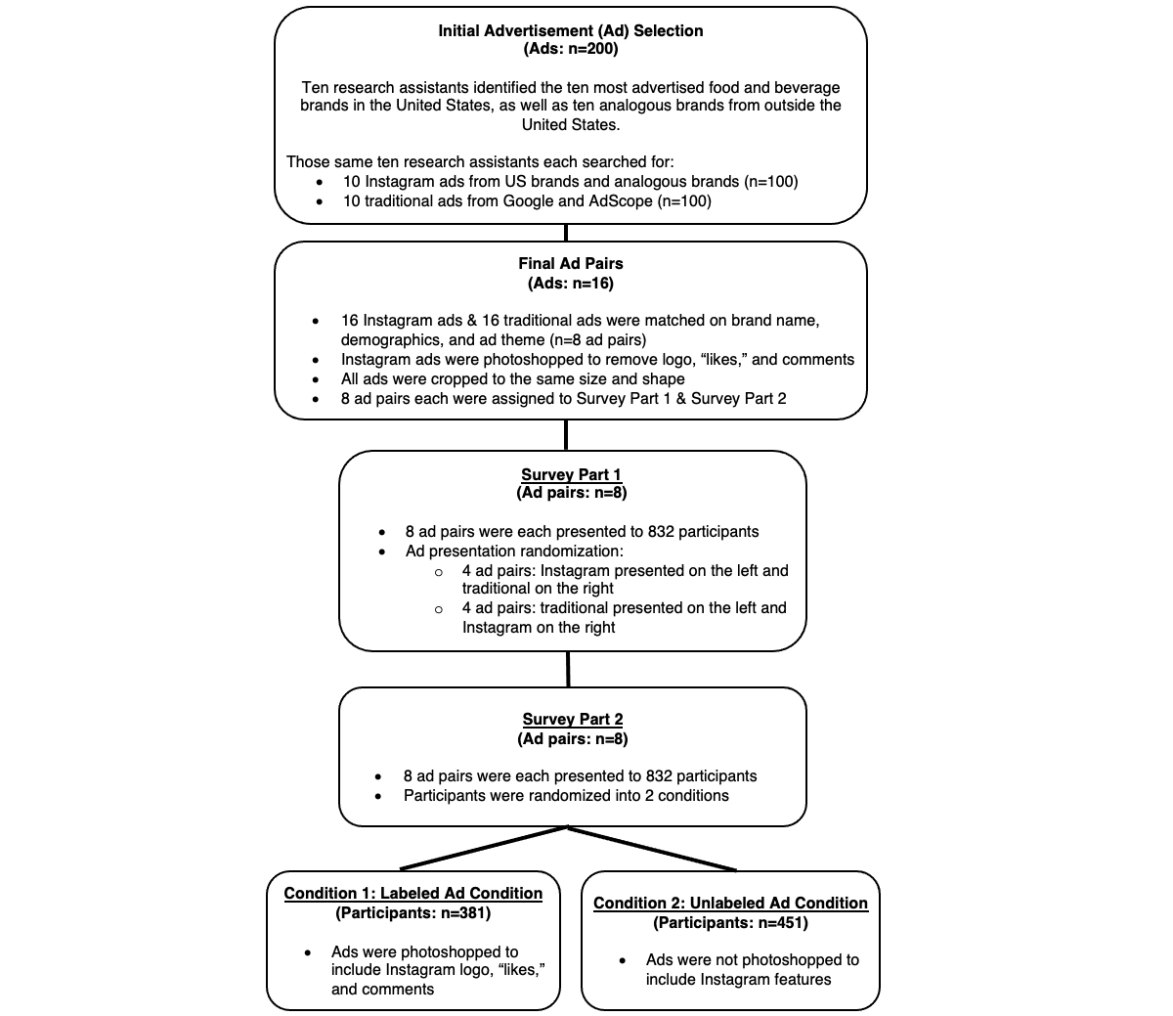
Advertisement development process and study procedure.

Researchers came to unanimous consensus on all matched pairs between traditional and Instagram ads based on demographic characteristics of the people in the ad and ad theme. We asked the researchers to identify 10 of the final 16 ad pairs from brands originating outside of the United States. By using ads from brands based in the United States we could evaluate reactions to ads that the participants would be most likely to see in their day-to-day life. By including some brands based outside of the United States, we could isolate reactions to the ad-specific effects of interest from potential confounding factors of preformed attitudes toward brands. As intended, this search process generated a final set of 32 unhealthy food and beverage ads, which included 16 ad pairs that were matched on brand name, demographics, and ad theme (Figure 1).

### Survey Procedures

After parents provided informed consent, adolescents assented their participation and completed the online survey with a median completion time of 19 minutes. Qualtrics hosted the online survey. Data were collected in 2018 and analyzed in 2019.

**Figure 2:**
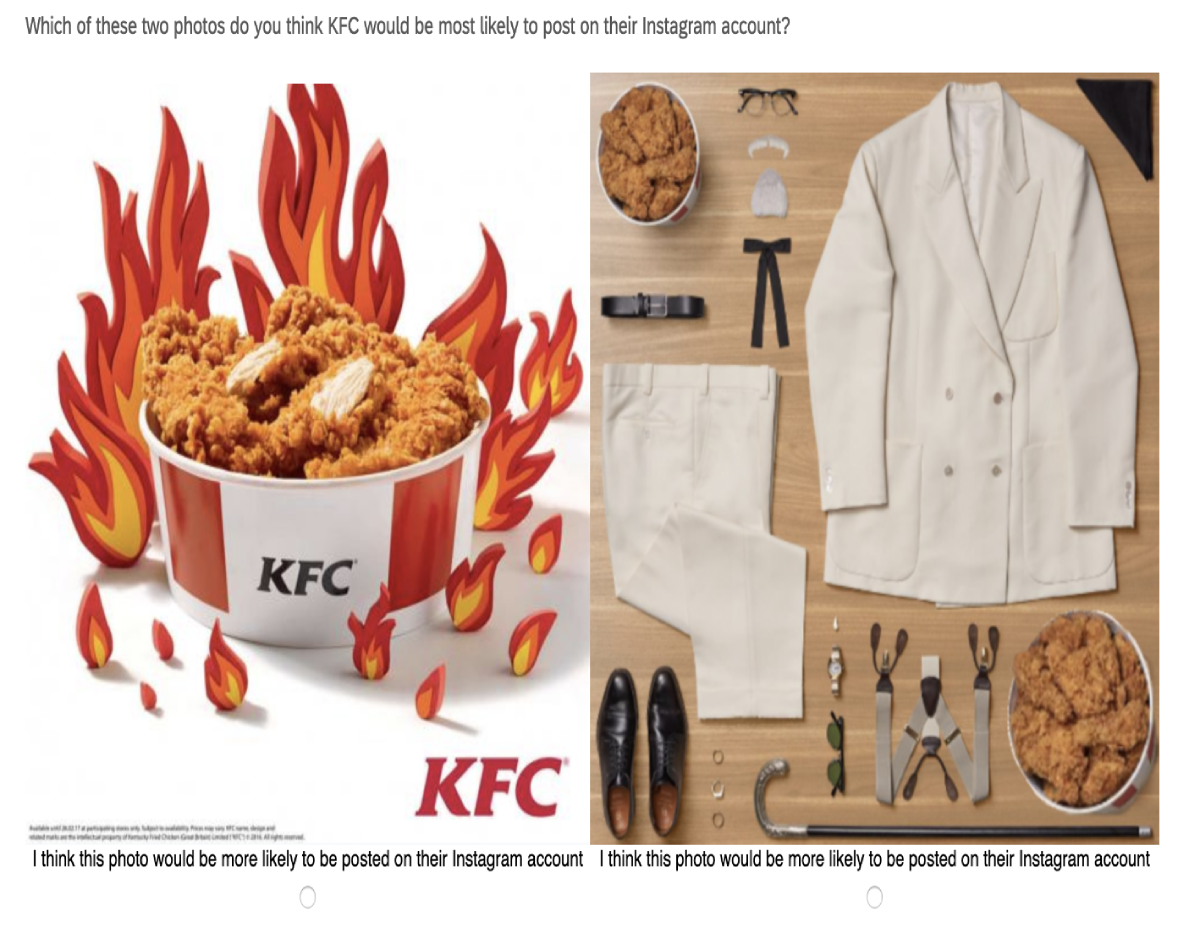
Sample Question from Part 1: Identify the Instagram ad. In this question, the Instagram ad is on the right.

In Part 1 of the survey, adolescents viewed 8 pairs of unhealthy food and beverage ads presented in random order (eg, a Starbucks magazine ad alongside a Starbucks Instagram ad). To remove cues indicating which ads originated from Instagram, we used Photoshop to compare advertising images with and without an “Instagram frame” which includes the Instagram logo, “likes,” and comments. We also cropped images to make ads the same shape because Instagram images are often square, which would have made it easier for adolescents to identify the Instagram ad. See Figure 2 for an example of our ad stimuli. We randomized the ad presentation so that half the pairs showed the Instagram ad on the left and the traditional ad on the right (Figure 1).

After viewing each pair, adolescents answered the following question, “Which of these photos do you think [brand name] would be most likely to post on their Instagram account?” See Figure 2 for an example of survey questions and images from Part 1.

**Figure 3:**
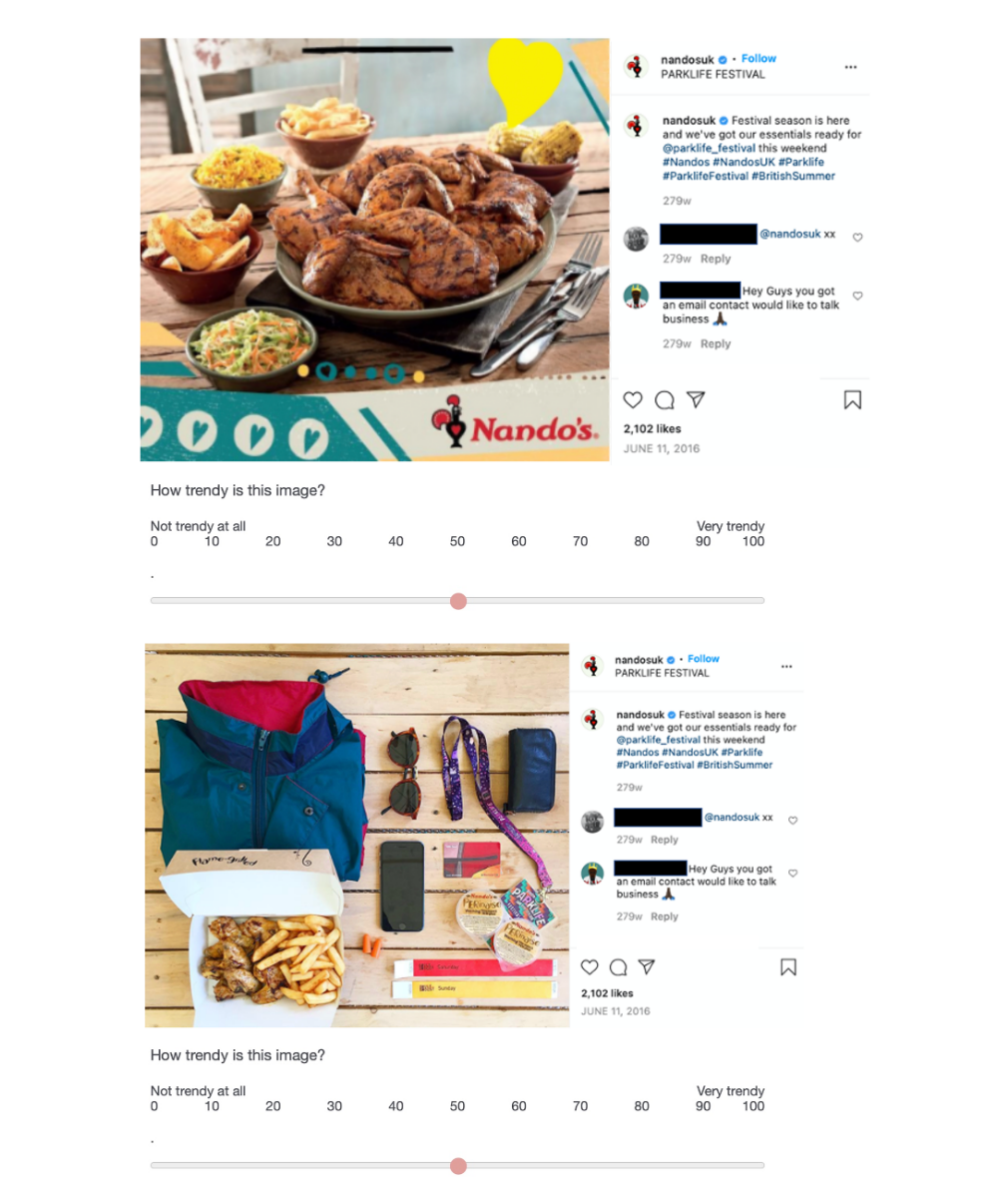
Sample images from “Labeled Ad Condition.” The traditional ad (top) and the Instagram ad (bottom) both feature the Instagram panel.

**Figure 4:**
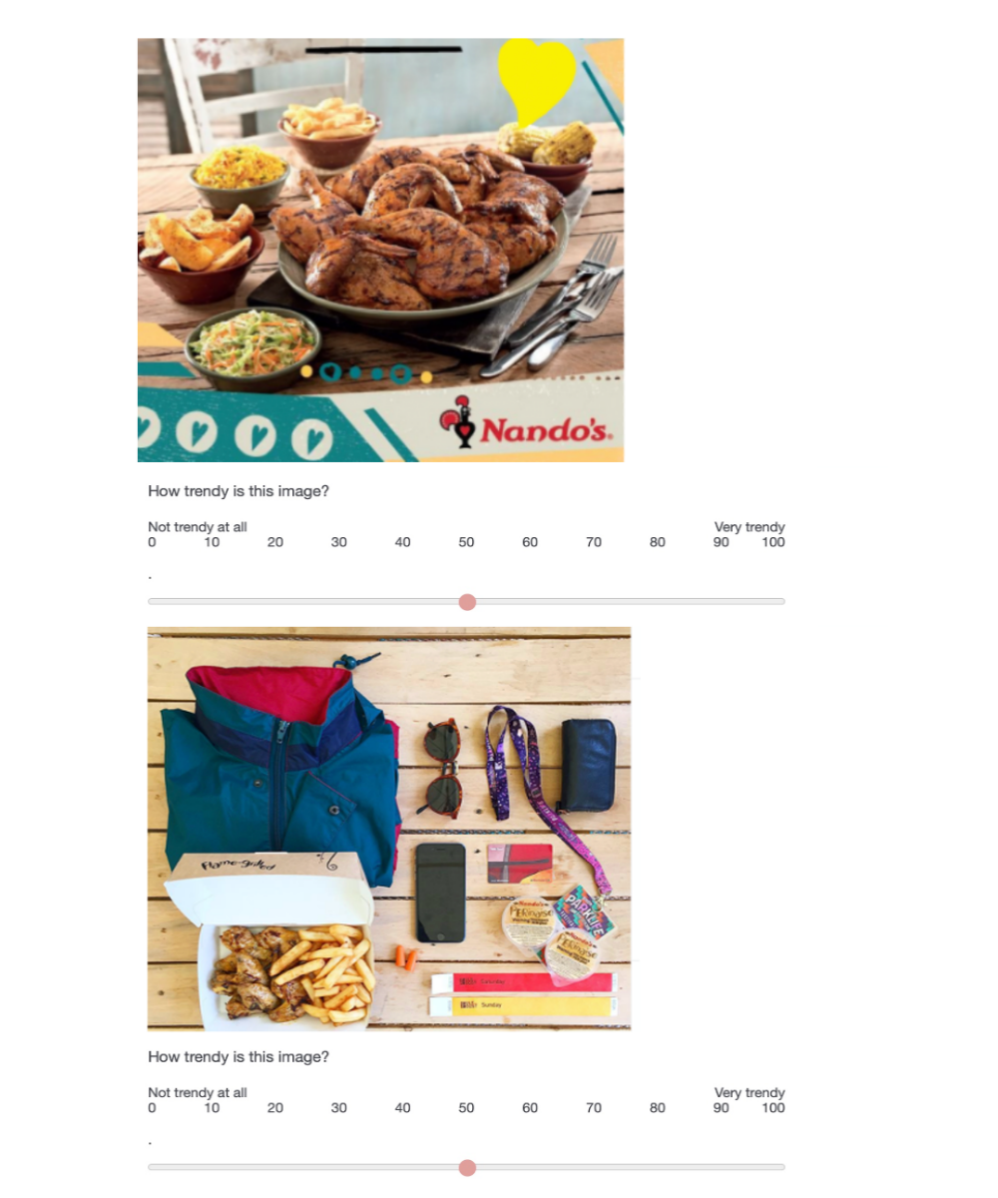
Sample images from “Unlabeled Ad Condition.” The traditional ad (top) and the Instagram ad (bottom) do not feature the Instagram panel.

Adolescents were then randomly assigned to 1 of 2 conditions (Part 2) where ads ostensibly originated from (1) Instagram (ie, “labeled ad condition” in which advertising images have Instagram frames); or (2) traditional sources (ie, “unlabeled ad condition” in which advertising images do not have Instagram frames; Figure 1). Unbeknownst to participants, half of the ads in their condition originated from Instagram and half originated from traditional sources. Ads were presented in random order. The survey questions asked participants to rate the ad on how much they liked the image, trendiness, artistic appeal, how delicious they thought the featured product might be, and how likely they were to purchase the product (Table 2). We included the ratings of trendiness and artistic appeal because previous commentaries have noted that social media food ads are subtle and blend into the social media environment [10,26]. See Figures 3 and 4 for examples of survey questions and images from Part 2. Finally, participants responded to demographic questions. We included an attention check question (ie, “Type Facebook in the box”) to ensure participants were carefully reading directions and questions. Those who did not type “Facebook” were excluded from analysis.

**Table 2:** Instagram and traditional advertisement ratings and differences by study condition, means, and SEs.

Participant advertisement ratings		Average rating of Instagram advertisement (SE)^a^	Average rating of traditional advertisement (SE)	Difference in ratings (SE)	*P* values^b^
**Unlabeled advertisement condition, rating out of 0-100^c^ (SE)**					
	How much do you like this image?	68.56 (0.93)	65.72 (0.93)	2.85 (0.49)	.001^d^
	How artistic is this image?	68.83 (0.93)	66.86 (0.93)	1.97 (0.47)	.001^d^
	How trendy is this image?	69.60 (0.90)	66.28 (0.90)	1.14 (0.54)	.001^d^
	How delicious do you think this product is?	66.80 (0.94)	65.66 (0.94)	1.14 (0.54)	.07
	How likely are you to purchase this product in the next 4 weeks?	56.25 (1.23)	55.27 (1.23)	0.97 (0.58)	.10
**Labeled advertisement condition, rating out of 0-100^c^ (SE)**					
	How much do you like this image?	67.43 (1.04)	67.11 (1.04)	0.32 (0.50)	>.99
	How artistic is this image?	66.80 (1.07)	66.84 (1.07)	0.04 (0.50)	>.99
	How trendy is this image?	68.16 (1.02)	66.92 (1.02)	1.24 (0.48)	.05^d^
	How delicious do you think this product is?	66.62 (1.02)	67.51 (1.02)	0.88 (0.53)	.40
	How likely are you to purchase this product in the next 4 weeks?	56.03 (1.38)	56.30 (1.38)	0.27 (0.60)	>.99

^a^SE: standard error.

^b^All *P* values are corrected for multiple comparisons using the Bonferroni–Holm procedure.

^c^Ratings were based on a scale from 0 (not at all) to 100 (very much).

^d^Statistical significance (*P*<.05).

### Statistical Analyses

Analyses were conducted in SAS 9.4 (SAS Institute). We used a score test to assess whether the proportion of correct responses was significantly different from 0.5, which is the percentage of correct responses we would expect if adolescents were guessing at random. We also used linear regression to compare adolescents’ ad preferences between the unlabeled ad condition and the labeled ad condition. Ad preferences were scored from 0 to 100. Because each adolescent rated multiple ads, the model included a random effect for the participant to account for the repeated measures. Given adolescents spend approximately 7 hours per day using screens [27], it is critical to understand the extent to which heavy use of social media is associated with higher preferences for social media food ads compared with traditional food ads. To examine differences based on time spent on social media, we analyzed data according to time spent on social media. Specifically, analyses that adjusted for time spent on social media used a median split at 3 hours, such that adolescents who reported spending more than 3 hours per day on social media were labeled “heavy social media users” and those who reported spending less than 3 hours per day on social media were labeled “light social media users.” Analyses were then stratified by whether the ad had the “Instagram panel.” We conducted chi-square tests and *t* tests to determine whether the randomization was successful and to verify that demographic characteristics did not differ between conditions. Because all tests were not significant, demographic characteristics were not included in the models. The Bonferroni–Holm procedure was used to correct for multiple comparisons.

## Results

### Part 1: Adolescents Were Asked to Identify Which Advertisements Originated from Instagram

Adolescents correctly identified the Instagram ad 39.1% of the time (*z*=17.293, *P*<.001). They correctly selected the Instagram ad significantly more often for international brands (42.8% of the time) than US brands (35.3% of the time; *z*=36.55, *P*<.001). We did not find a significant association between the likelihood of adolescents following the US brands on social media and their ability to accurately identify the Instagram ads for unhealthy food and beverages (*z*=.0022, *P*=.96), suggesting that the association was not confounded by brand familiarity.

In an analysis stratified by time spent using social media, we found that both light and heavy social media users performed worse than chance (*z*=15.79, *P*<.001 and *z*=8.4387, *P*<.001). However, heavy social media users correctly identified the Instagram ad more often (42.2%) than light users (36.3%; *z*=22.69, *P*<.001).

### Part 2: Adolescents Rated Preferences for Instagram Advertisements and Traditional Advertisements

In the “unlabeled ad condition” where all ads for unhealthy products were presented without any Instagram features (Figure 4), analyses revealed adolescents reported higher preferences for Instagram ads than traditional ads on 3 of 5 measures after correcting for multiple comparisons (Table 2). Specifically, adolescents rated Instagram ads for unhealthy food and beverages as significantly trendier (*P*=.001) and more artistic (*P*=.001) than the traditional ads. They also reported liking the Instagram ads more than the traditional ads for unhealthy products (*P*=.001). There were no significant differences in how delicious they thought the featured products might be (*P*=.08) or how likely they would be to purchase them (*P*=.10).

Within the “labeled ad condition,” we found significant differences on 1 of the 5 outcomes (Figure 3). Specifically, adolescents were more likely to rate the unhealthy food and beverage ads on Instagram as trendy compared with the traditional ads (*P*=.05), but they rated the ads similarly on the other 4 dimensions.

Heavy social media users responded more positively to social media ads for unhealthy food and beverages than light users across all 5 outcomes in both the “labeled ad condition” and the “unlabeled ad condition.” In the “ labeled ad condition,” heavy social media users rated ads as significantly more artistic (9.46 points; standard error [SE] 2.02, *P*<.001), trendier (9.30 points; SE 1.93, *P*<.001), and delicious (7.69 points; SE 1.94, *P*<.001); they also reported liking ads more (9.37 points; SE 1.98, *P*<.001) and being more likely to purchase the featured products (15.84 points; SE 2.57, *P*<.001). In the “unlabeled ad condition,” heavy social media users rated unhealthy food and beverage ads as more artistic (8.01 points; SE 1.76, *P*<.001), trendy (8.20 points; SE 1.70, *P*<.001), and delicious (7.92 points; SE 1.77, *P*<.001). They also reported liking them more (7.73 points; SE 1.76, *P*<.001) and being more likely to purchase the products pictured (13.68 points; SE 2.31, *P*<.001).

## Discussion

### Principal Findings

Our findings demonstrate that Instagram food ads were highly appealing to adolescents relative to traditional food ads. This is concerning given adolescents spend 7 hours online each day [27], and companies now spend US $41.5 billion on social media marketing each year [28]. Coca-Cola alone spends US $800 million—20% of their US $4 billion budget—on social media marketing [3], suggesting that companies may be increasingly able to place more ads in front of adolescents in digital spaces. Adolescents incorrectly identified traditional ads as Instagram ads, consistently, suggesting that the artistic quality of Instagram ads may not be perceived as marketing. The subtlety of these Instagram ads may create a public health challenge because adolescents might be more vulnerable to the persuasive influence of these visually artistic and entertaining ads. Additionally, when rating unlabeled, unhealthy food and beverage ads in Part 2, adolescents reported higher preferences for Instagram ads compared with traditional ads on 3 of 5 outcomes. This finding suggests that there is something uniquely appealing about the visual appearance of Instagram ads compared with traditional ads that piques adolescents’ interests. But, when we photoshopped the Instagram frame onto those same ads, adolescents preferred the Instagram ads on just 1 of 5 outcomes. This suggests that the “Instagram frame” exerts a powerful influence on adolescents’ perceptions by equalizing the appeal of traditional and Instagram ads for unhealthy products. In 2019, 2 bipartisan US Senators proposed expanding the Children’s Online Privacy and Protection Act to reduce companies’ ability to target youth in online advertising—their proposal would also extend the protected age range to include adolescents aged 13-16 years [29]. Providing policy protections for adolescents in digital spaces is critical given Instagram food ads are highly appealing to adolescents compared with traditional ads.

Contrary to our hypothesis that adolescents would be skilled at identifying which of 2 ads originated from Instagram in Part 1, adolescents performed significantly worse than chance by incorrectly choosing the traditional ad. One possibility is that adolescents could not discern between ads that originate on Instagram and traditional outlets—but adolescents’ responses were worse than 50-50 chance, meaning they *consistently* thought the traditional ad was an Instagram ad. One explanation for the recurring misattribution is that companies mimic social media trends that are highly appealing to adolescents (eg, photo filters or aerial photos of foods arranged in artistic ways), and such mimicry is not readily perceived as marketing [30-32]. These findings support that ads promoted on Instagram are highly appealing to adolescents compared with ads presented in a more traditional form.

The findings that “heavy users” reported higher ad preferences across all outcomes—and were more likely to “guess correctly” in Part 1 relative to “light users”—may be concerning because of the potential additive effect of social media: when users “like” posts, Instagram’s algorithms place similar posts in their feed, thereby increasing ad exposure [33]. It is possible, however, that “heavy users” merely have more Instagram fluency, which may have accounted for their ability to correctly identify the Instagram ads. And it is possible that their heavy use of media makes them more inclined to like any type of image that stems from Instagram or other outlets.

This study contributes to the literature on food marketing in several ways. It is the first study, to our knowledge, to compare unhealthy food ads on social media with traditional ads. The power of the Instagram “halo” is concerning given adolescents’ exposure to food ads on social media—one study found that 72% (n=101) of adolescents were exposed to social media food ads during a 5-minute data collection period [34], and another survey of 1564 adolescents in the United States found that 75% of adolescents followed, “liked,” or shared food and beverage brands on social media [35]. This “halo” effect has also been shown to impact children and adolescents’ eating behaviors. In a laboratory study, children who viewed influencers holding unhealthy snacks ate significantly more food than children who saw influencers holding nonfood products [36]. Another laboratory study randomized 132 adolescents aged 13-16 years to view a social media influencer who promoted unhealthy food, vegetables, or a nonfood item and found that adolescents exposed to the healthy food posts did not consume more vegetables [37]. Finally, one study of 72 adolescents (mean age 13 years) found that adolescents who viewed unhealthy food brand posts were more willing to share the post and report a positive attitude toward the product compared with adolescents who viewed healthy foods [38]. But one limitation of existing social media food ad studies is the absence of nonsocial media ads. Our study, therefore, builds upon previous research by demonstrating that the mere presence of the Instagram logo, “likes,” and comments causes adolescents to rate ads more favorably than adolescents who see the same ads without those Instagram features.

### Limitations

This study has several limitations. We did not ask participants about their rationale for choosing the traditional ad or the Instagram ad in Part 1, and additional research is needed to determine whether differences between heavy and light users’ ratings translate into increased susceptibility to advertising. We did not collect data on self-reported height and weight, and it is possible that BMI could moderate the observed effects [39]. But studies suggest that BMI does not always predict higher preferences for ads [40,41]. Our study may not generalize to all adolescents given this was not a nationally representative sample. Despite these limitations, our study has several strengths. The randomized design and the within-subjects comparisons provide clear evidence of preferences for unhealthy food and beverage ads on Instagram relative to traditional ads. Further, “likes” are an ecologically valid signal of preferences, as most adolescents use social media regularly and are familiar with “liking” social media content [42]. Future research should examine how social media food ads affect food purchases.

### Conclusion

This study provides the first evidence regarding adolescents’ preferences for unhealthy food and beverage ads on social media relative to traditional food ads, sheds light on potential mechanisms that influence adolescents’ behavior on social media, and can inform the extent to which self-regulatory food marketing pledges and food marketing policies should expand to include social media–based food marketing.

## Abbreviations

ads: advertisements
SE: standard error
